# Editorial: Bacteriophages and Their Lytic Enzymes as Alternative Antibacterial Therapies in the Age of Antibiotic Resistance

**DOI:** 10.3389/fmicb.2022.884176

**Published:** 2022-03-25

**Authors:** Carlos São-José, Ana Rita Costa, Luís D. R. Melo

**Affiliations:** ^1^Research Institute for Medicines (iMed.ULisboa), Faculty of Pharmacy, University of Lisbon, Lisbon, Portugal; ^2^Department of Bionanosciences, Kavli Institute of Nanoscience, Delft University of Technology, Delft, Netherlands; ^3^CEB – Centre of Biological Enginering, LIBRO-Laboratório de Investigação em Biofilmes Rosário Oliveira, University of Minho, Braga, Portugal; ^4^LABBELS – Associate Laboratory, Braga/Guimarães, Portugal

**Keywords:** phage therapy, lysins, antibiotic resistance, enzybiotics, bacteriophage (phage)

The misuse and abuse of antibiotics observed in the past decades have accelerated the evolution of antibiotic-resistant bacteria, which led us to the brink of a post-antibiotic era where trivial bacterial infections become life-threatening (Levy and Marshall, 2004). Now, health organizations worldwide are urgently calling for the development of new strategies against antibiotic-resistant bacteria and the spread of resistance (IACG, 2019). In this context, bacteriophages and their lytic enzymes (lysins) are a definite route to explore.

The use of bacteriophages to treat bacterial infections has been common in Eastern Europe over the past century (Zaczek et al., 2020), and is now also considered an alternative to antibiotics in the Western world, supported by a number of successful case reports (reviewed in Hatfull et al., 2022). Lysins of bacteriophages represent a different approach in the fight against antibiotic-resistant bacteria. These lytic enzymes cleave the bacterial cell wall peptidoglycan and were initially considered particularly suited to attack the exposed cell wall of Gram-positive (G+) bacteria (Murray et al., 2021). Lysin application as enzybiotics was meanwhile expanded to Gram-negative (G–) bacteria with the development of strategies that overcome the physical barrier imposed by the outer membrane (OM) in these bacterial cells (reviewed in Gutierrez and Briers, 2021). A few lysins targeting *Staphylococcus aureus* are currently in different phases of clinical trials (summarized in Danis-Wlodarczyk et al., 2021).

This Research Topic aimed at providing a platform for sharing the most recent advances on the field of phage-derived antimicrobials for control and prevention of antibiotic-resistant bacterial infections.

In the past 2 years, the SARS-CoV-2 pandemic challenged the world and contributed further to the antibiotic resistance crisis, due to the prophylactic administration of antibiotics to prevent secondary bacterial infections (Feldman and Anderson, 2021). In this Research Topic, Alsaadi et al. discuss the importance of learning from mistakes in past pandemics and prevent future health problems caused by multidrug resistant (MDR) bacteria by investing in R&D of phage therapy. This perspective article further discusses the possibility of engineering phage genomes to improve their potential, and highlights the need for a phage-based regulatory framework and for raising awareness in society about phage therapy.

Despite phages being generally described as specific for bacteria and with no negative interactions with eukaryotic cells, some reports suggest that phages can affect the metabolism of mammalian cells (Merril, 1973). In this Research Topic, Górski et al. elaborate about the therapeutic implications of phage interactions with epithelial cells. The authors discuss the ability of phages to bind and penetrate different types of mammalian cells and to alter their phenotype (e.g., alterations of expression of surface markers and immunomodulation of cytokine production). As small doses of pathogen-derived molecules might elicit immune response the authors highlight the importance of administering highly pure phages in therapy.

A widely discussed drawback of phage therapy is the ability of bacteria to quickly develop resistance to phages. This can be circumvented with the use of phage cocktails (Chan et al., 2013). To develop an efficient cocktail, Niu et al. systematically evaluated the interaction of four phages in single therapy and in 11 cocktails for biocontrol of *Escherichia coli* O157. Variables such as multiplicity of infection, temperature, and exposure times were all found to influence interactions among phages. Importantly, the authors detected several antagonistic interactions between phages, and therefore suggest that phage cocktail formulation should guarantee absence of antagonistic interactions and favor synergistic effects.

A growing body of evidence supports that several lysins have an intrinsic capacity to cross the OM, thanks to features more frequently found on their C-termini (concentration of positively charged amino acids and/or presence of AMP-like segments; Vazquez et al., 2021). Vásquez et al. show that the bioinformatics analysis of lysin physicochemical properties coupled to a predictive *k*-nearest neighbors model can be used for “mining” putative lysins with these membrane-interacting features. The intrinsic bactericidal activity against G- bacteria was demonstrated for two lysin candidates emerging from the analysis, validating the approach. This work provides a new venue to uncover and eventually engineer novel lysin-based antimicrobials with enhanced features.

Recently, the Lin lab showed that lysins produced by phages from thermophilic environments can have bactericidal activity both against G- and G+ bacteria (Wang et al., 2020a,b). In this Research Topic, the same group reports the case of P9ly, a lytic enzyme produced by the *Shigella dysenteriae* infecting phage PSD9 that has the same capacity. Wang et al. show that besides being bacteriolytic toward different *Shigella* species and other G- bacteria like *E. coli* and *Salmonella* spp., P9ly can also lyse a MDR *S. aureus* strain. This study provides preliminary data pointing to an enhanced bactericidal effect resulting from the P9ly/antibiotic combined action. Of note, P9ly adds to the very few lysins with bactericidal action on *Shigella* spp. reported to date (Deng et al., 2021; Xu et al., 2021).

Anti-streptococcal endolysins are amongst the best characterized phage lytic enzymes acting on G+ bacteria and were the first shown to efficiently kill bacterial pathogens *in vivo* (Loeffler et al., 2001; Nelson et al., 2001). Gallego-Páramo et al. report in this Research Topic a detailed structural and functional characterization of two streptococcal endolysins, Skl and Pal. By performing deep computational modeling of the endolysins' functional domains, and supported by extensive physical, genetic and biochemical analysis, the authors reveal key molecular details of the enzymes regarding substrate recognition, catalytic mechanism and 3D structure. Skl and Pal, which have distinct catalytic (CHAP and Amidase_5 families, respectively) and related cell binding domains, share a similar fold. Also shared between these endolysins and other known streptococcal lytic enzymes is the overall 3D architecture observed upon dimerization promoted by choline binding. This suggests an adaptation of streptococcal lysins to choline-containing cell walls.

Overall, this collection of six articles provides an excellent update in the field of bacteriophage-derived antimicrobials and formulates questions to be addressed by the scientific community worldwide.

## Author Contributions

All authors listed have made a substantial, direct, and intellectual contribution to the work and approved it for publication.

## Conflict of Interest

The authors declare that the research was conducted in the absence of any commercial or financial relationships that could be construed as a potential conflict of interest.

## Publisher's Note

All claims expressed in this article are solely those of the authors and do not necessarily represent those of their affiliated organizations, or those of the publisher, the editors and the reviewers. Any product that may be evaluated in this article, or claim that may be made by its manufacturer, is not guaranteed or endorsed by the publisher.
